# Tuberculosis Infection: Occurrence and Risk Factors in Presumptive Tuberculosis Patients of the Serengeti Ecosystem in Tanzania

**DOI:** 10.24248/EAHRJ-D-16-00319

**Published:** 2017-03-01

**Authors:** Erasto V Mbugi, Bugwesa Z Katale, Athumani M Lupindu, Julius D Keyyu, Sharon L Kendall, Hazel M Dockrell, Anita L Michel, Mecky I Matee, Paul D van Helden

**Affiliations:** a Department of Biochemistry, Muhimbili University of Health and Allied Sciences, Dar es Salaam, Tanzania; b Departments of Microbiology and Immunology, Muhimbili University of Health and Allied Sciences, Dar es Salaam, Tanzania; c Tanzania Wildlife Research Institute, Arusha, Tanzania; d Department of Veterinary Medicine and Public Health, Sokoine University of Agriculture, Morogoro, Tanzania; e The Royal Veterinary College, London, United Kingdom; f Department of Immunology and Infection, London School of Hygiene and Tropical Medicine, London, United Kingdom; g Department of Veterinary Tropical Diseases, University of Pretoria, Pretoria, South Africa; h DST/NRF Centre of Excellence for Biomedical Tuberculosis Research/Medical Research Council, Centre for Molecular and Cellular Biology, Stellenbosch University, Tygerberg, South Africa

## Abstract

**Background:**

Cross-species tuberculosis (TB) transmission between humans and animals has been reported for quite a long time in sub-Saharan Africa. Because humans and animals coexist in the same ecosystem, exploring their potential for cross-species transmission and the impact the disease may have on the health of humans, animals, and their products is critical.

**Objectives:**

This study aimed to identify risk factors for transmission of TB (*Mycobacterium tuberculosis*) and to assess the potential for zoonotic TB (*Mycobacterium bovis*) transmission in the Serengeti ecosystem where humans and animals are in intense contact. Our aim is to create a base for future implementation of appropriate control strategies to limit infection in both humans and animals.

**Methodology:**

We administered a semi-structured questionnaire to 421 self-reporting patients to gather information on risk factors and TB occurrence. In a parallel study, researchers screened sputum smears using Ziehl–Neelsen staining and confirmed by mycobacterial culture. We then performed descriptive statistics (Pearson's chi-square test) and logistic regression analysis to establish frequencies, association, and quantification of the risk factors associated with TB cases.

**Results:**

Our findings showed 44% (95% confidence interval [CI], 0.40-0.49) of the results were positive from sputum samples collected over a 1-year duration in areas with a high TB burden, particularly the Bunda district, followed by the Serengeti and Ngorongoro districts. Of the culture-positive patients who also had infections other than TB (43/187 patients), 21 (49%) were HIV positive. Contact with livestock products (odds ratio [OR] 6.0; 95% CI, 1.81–19.9), infrequent milk consumption (OR 2.5; 95% CI, 1.42–4.23), cigarette smoking (OR 2.9; 95% CI, 1.19–7.1.2), and alcohol consumption (OR 2.3; 95% CI, 1.22–4.23) were associated with a higher likelihood of TB infection.

**Conclusion:**

There was no evidence of direct cross-species transmission of either *M tuberculosis* or *M bovis* between humans and animals using the study methods. The absence of cross-species TB transmission could be due to limited chances of contact rather than an inability of cross-species disease transmission. In addition, not all people with presumptive TB are infected with TB, and therefore control strategies should emphasise confirming TB status before administering anti-TB drugs.

## INTRODUCTION

The incidence and prevalence of human tuberculosis (*Mycobacterium tuberculosis* [TB]) in Tanzania is not adequately determined due to absence of regular screening. This is despite the reported estimate incidence rate of 164 per 100,000 persons per year and a prevalence of 0.4%.^1^ In animals, particularly cattle, no national TB surveys have been performed; however, localised studies report the prevalence of bovine TB to range from 0.2% to 13.3%.^2^ Reports from some parts of northern Tanzania indicated the presence of TB in humans caused by zoonotic TB (*Mycobacterium bovis*), which is often characterised by TB adenitis features.^3,4^ In a separate study, Cleaveland and colleagues^5^ reported the potential risk factors for infection by *M bovis* for humans and cattle in rural Tanzania, and provided possible evidence of a link between infections among humans, cattle, and wildlife where they live in close proximity. Despite reports from the Horn of Africa that *M bovis* makes only a minor contribution to human TB,^6,7^ the potential for *M bovis* to infect humans may be significant in some groups, such as pastoral communities, where TB can be acquired through the air (pulmonary TB) and through consumption of contaminated animal products. For example, in 2007 Shitaye and colleagues^8^ reported prevalence of TB in cattle ranging from 3.4% (in smallholder production systems) to 50% (in intensive dairy productions) and 3.5% to 5.2% in slaughterhouses in Ethiopia. This level of prevalence is concerning, particularly in areas where humans and animals are in intense contact like in the Serengeti ecosystem. The contribution of *M bovis* to human TB is potentially 37.7% of all human TB cases in Africa.^9^ In Tanzania the median contribution of *M bovis* in human TB is 26.1% (range 10.8%–37.7%), further increasing concerns about zoonotic TB with regional variations.

Given the challenges of laboratory TB diagnosis in resource-poor countries,^10^ identification of hotspots or at-risk communities could help direct resources, such as laboratory tests, therapy, or intervention programmes, and make them more cost-effective. Directing more resources to communities at risk may be useful in determining the spread of TB in areas where humans and animals coexist, exploring their potential for cross-transmission, and understanding the impact the disease may have on the health of humans, animals, and their products.

We conducted the study by administering a semi-structured questionnaire in health centres in the Serengeti ecosystem to collect information about TB disease status and transmission. We administered the questionnaires in parallel with a different study that tested (using bovine tuberculin skin testing) for the presence of TB in cattle in the Serengeti ecosystem^11^ before collecting samples from TB-infected humans and animals. Animal tissues from cattle with presumptive TB and archived tissues with presumptive TB from wildlife were then cultured and mycobacterial DNA characterised to identify circulating TB strains in the ecosystem. These results, published separately, showed no *M tuberculosis* strains in animals indicative of no potential for at least *M tuberculosis* to infect animals.^12^ These findings ruled out potential for zoonotic tuberculosis (M bovis) transmission,^13^ initially thought to be the case to create a base for future joint implementation of appropriate control strategies to limit infection in both humans and animals.

## METHODS

### Study Site

**FIGURE 1.**
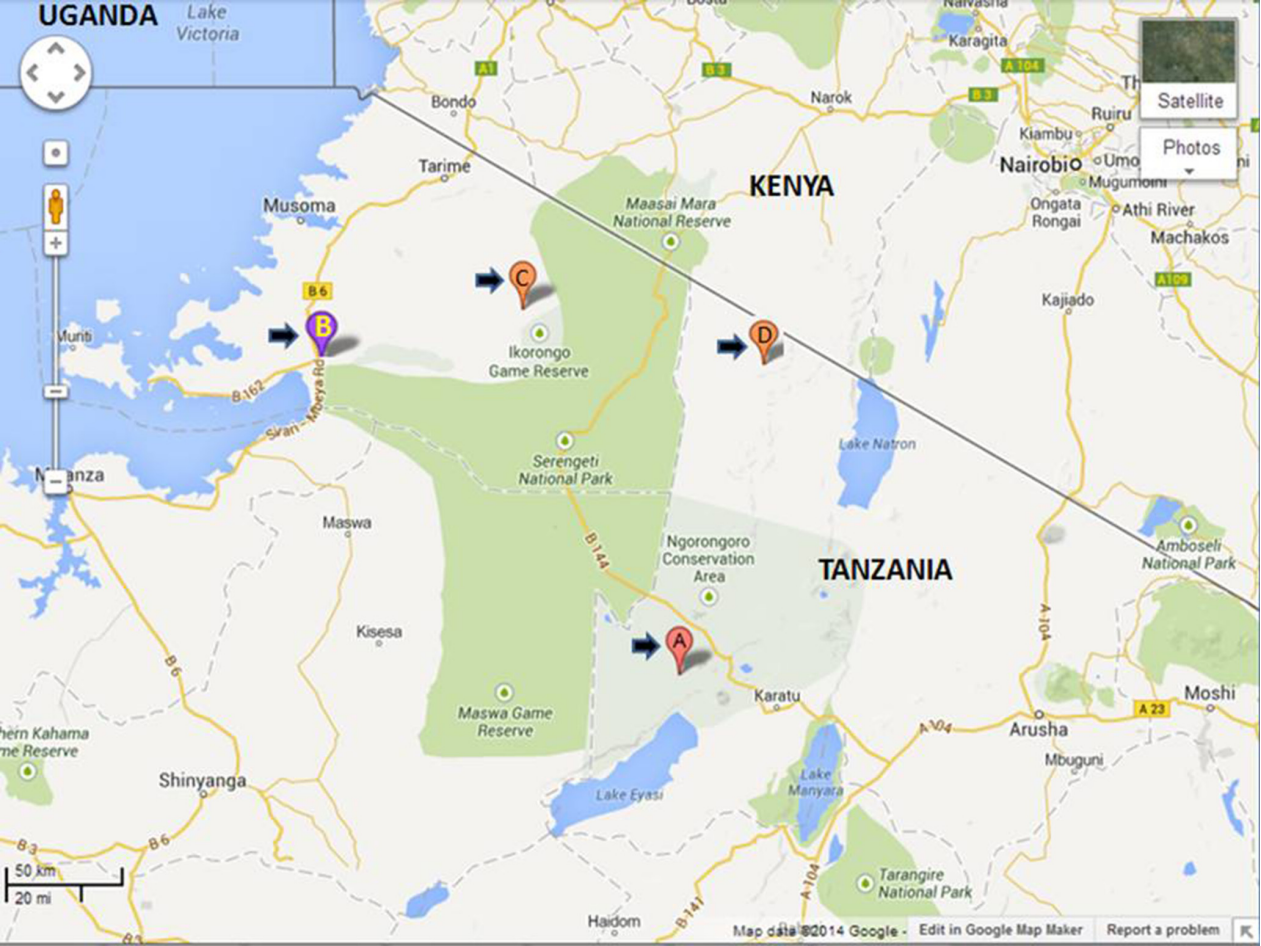
Map of Health Centres Within the Study Site (Serengeti Ecosystem)

The semi-structured interview was carried out between October 2010 and November 2012 in health centres in the Serengeti ecosystem. These centres were Endulen Health Centre (in the Ngorongoro Conservation Area), Bunda district-designated hospitals (serving villages within the district and from neighbouring districts), Mugumu district-designated hospital (in Serengeti District), and Waso district-designated hospital (in Ngorongoro) (Figure 1). The centres were selected because they serve as focal points where TB screening is performed regularly.

A: Endulen Heath Centre; B: Bunda district-designated hospital; C: Mugumu district-designated hospital; D: Waso district-designated hospital.

### TB Testing Methods

As described earlier, sputum smear samples were collected from self-reporting participants (all age groups) with presumptive TB and then tested using Ziehl–Neelsen staining, microscopic examination for acid-fast bacilli, and mycobacterial culture. At the time of sample collection, we administered paper-based semi-structured questionnaires to participants to collect information about TB disease status and transmission in these ecosystems. Our testing methods differed from other groups^2–5^ who have conducted TB studies in Tanzania with a focus on either humans or animals. The sampling in this study was at the interface of intense human-animal contact. In such interfaces, the possibility of cross-species transmission might be comparatively high due to a heightened interaction.

### Participant Criteria

Inclusion criteria for participating in the questionnaire consisted of both inpatients and outpatients at health centres within the study sites who had symptoms suggestive of pulmonary TB (eg, prolonged recurrent fever, cough, anorexia, night sweating, weight loss, and general ill health of unknown cause) and who had not yet been on an anti-TB regimen. Patients who were already undergoing TB therapy were not eligible to participate in the study to avoid interference with study analysis outcomes. We understand that without symptomatic inclusion criteria likely to identify cases of *M bovis*, the study is limited; however, we believe that the results from the questionnaire provide an excellent overview regarding the dynamics of the disease in the course of interaction between humans and animals.

### Observations About the Study Population

**FIGURE 2.**
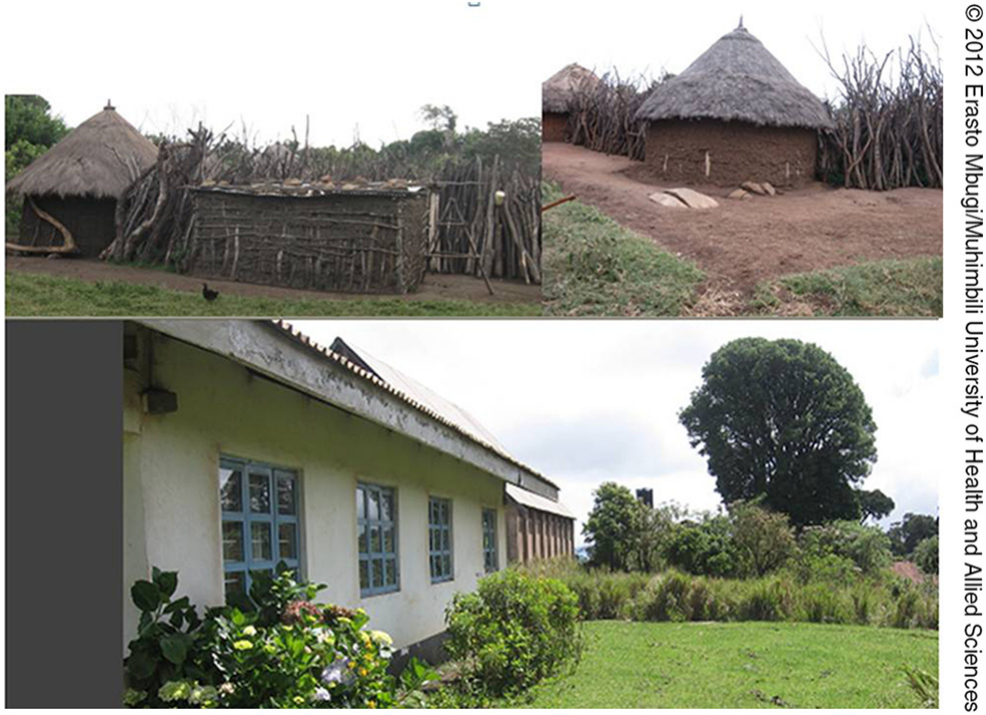
Different Types of Houses Within the Study Site (Serengeti Ecosystem)

The study population came from diverse home environments, ranging from modern houses to African traditional huts with different thatching and wall construction materials. Iron sheets, thatch grasses, and soil were commonly used as roofing materials, whilst walls were commonly made of cement bricks, mud, and poles with soil and animal dung in combination (traditional housing). These houses were also characterised by the number and size of windows, which affects ventilation and the potential for aerosol TB transmission in case there is a source of infection. We subjectively defined window sizes as small (approximately 30 cm by 30 cm or less), medium (approximately 60 cm by 45 cm), and large (exceeding the above sizes), based on information provided by respondents and our own experience. The pastoral societies live in clustered houses customarily known as a boma, which constitutes a household (Figure 2), with 2 or more houses that have enclosures for protection of animals from thieves and predators. This is particularly the case in Serengeti District and Ngorongoro Conservation Area.

In the top two pictures, the boma (clustered houses that constitute a household) represent traditional African huts with different roofing and construction materials, as well as windows (not shown). The clustered poles are an enclosure within the household where cattle are kept close to owners to prevent stock loss. The bottom picture represents a modern house with different kinds of windows.

### Administration of Questionnaire

At the time of sample collection, we administered a paper-based semi-structured questionnaire to acquire sociodemographic information that was considered useful to understand infection and transmission of TB in this ecosystem. The questionnaire was written in the common language (Swahili) and, where necessary, a translator familiar with the local language was used. The questionnaire was pre-tested in a pilot before the study began. Trained health workers administered the questionnaires and helped participants complete them at health centres during a face-to-face interview. In general, the questionnaire inquired about socioeconomic information, contact with animals and animal products, and household members’ health history including TB and HIV/AIDS status.

### Ethical Consideration

All patients enrolled in the study gave written informed consent. We obtained ethical clearance from the Muhimbili University of Health and Allied Sciences Research Ethics Committee (Ref.MU/PGS/PhD/R/Vol.1) and the National Institute for Medical Research (Ref. No. NIMR/HQ/R.8a/Vol. IX/1299). Participants received an explanation about the study purpose, what was expected of them, potential risks and benefits, confidentiality, and the voluntary nature of participation. Participants had a chance to ask questions before deciding whether to participate in the study or not. Patients with confirmed TB were offered treatment as stipulated in the Tanzania National Guidelines for Management of TB.^14^

### Sample Size Calculation

The sample size for this study was calculated based on the formula [n=(1.96)^2^ p (1−p)/d^2^] (where p=expected prevalence or proportion and d=precision) as suggested by Thrusfield^15^ at a 5% significance level and 5% precision using 0.172% as the estimated prevalence of TB in Tanzania.^1^ Based on this calculation, a minimum of 219 subjects was sufficient to be sampled and provide reliable information.

### Sample Collection, Storage, and Transport

When someone with presumptive TB presented for clinical evaluation by a medical practitioner at a health centre within the study site, instructions were given to collect 2 sputum samples: 1 collected early in the morning by the patient at home and 1 sputum collected on the spot at the health centre. From these specimens, sputum smears were prepared for microscopy on-site at the health facility, and the remaining sample was transported in cetylpyridinium chloride to the central TB laboratory in Dar es Salaam for mycobacterial culture and drug sensitivity testing.

### Sample Processing and Mycobacterial Culture

The Ziehl–Neelsen staining for acid-fast bacilli was performed according to existing standard protocols followed by mycobacterial culture on in-house prepared Löwenstein-Jensen medium containing glycerol (for *M tuberculosis*) or pyruvate (for *M bovis*).^12^ Quality control for Ziehl–Neelsen testing was achieved from the point of sample collection, which was done early in the morning at home by the patient and later at the health centre. When collection tubes were delivered to the patients, it was ensured that instructions for sample collection at home were clear and followed up with a demonstration. Ziehl–Neelsen testing was done on-site and later after the samples arrived at the laboratory in Dar es Salaam before culture. Drug susceptibility testing was performed according to standard proportion methods.^16^ The concentrations for each respective antibiotic tested were: isoniazid (1 μg/mL), rifampicin (40 μg/mL), ethambutol (2 μg/mL), and streptomycin (5 μg/mL). Species identification was performed by spacer oligonucleotide typing (spoligotyping), whose results have been reported separately.^12,17^

### Statistical Analysis

The research team analysed data using SPSS version 16.0 (SPSS Inc., Chicago, IL, USA). Descriptive statistics were used to examine the frequencies and associations between population attributes. Different household characteristics were compared using the Pearson Chi-square test at a significance level of 5% (*P*<.05). Logistic regression analysis was performed to quantify the risk factors associated with TB cases using SAS version 9.0 (SAS Institute Inc. Cary, North Carolina, USA). TB status was treated as a binary outcome variable. We analysed the following explanatory variables to examine their potential association with TB transmission: sex, age, marital status, number of household members, Bacillus Calmette–Guérin (BCG) vaccination history, other infections associated with TB, recent history of TB treatment, cattle keeping, recent contact with livestock and livestock products and nature of contact, milk drinking and frequency, milk boiling, sour milk drinking and frequency of use, source of milk, knowledge about TB, contact with TB patients, house roofing material, house construction material, the number of rooms in a house, the number and size of windows, sleeping in a household with livestock, cigarette smoking, alcohol consumption, visits to gatherings, visits to local brew centres, and the origin of people they meet during free social time.

Univariable analysis was run for each explanatory variable and a variable qualified for multivariable analysis when it had a likelihood ratio with a significance level less than 25% (*P*<.25). A multivariable model was built by a backward-stepwise regression, in which case the retention cut-off point was *P*<.05. Assessment of confounders was based on relative or absolute change of 25% or 0.1 respectively in coefficients of other variables. Model goodness of fit was assessed by a Hosmer-Lemeshow test, whereby a *P* value of more than .05 indicated that the model had a good fit.

## RESULTS

### General Results

**FIGURE 3.**
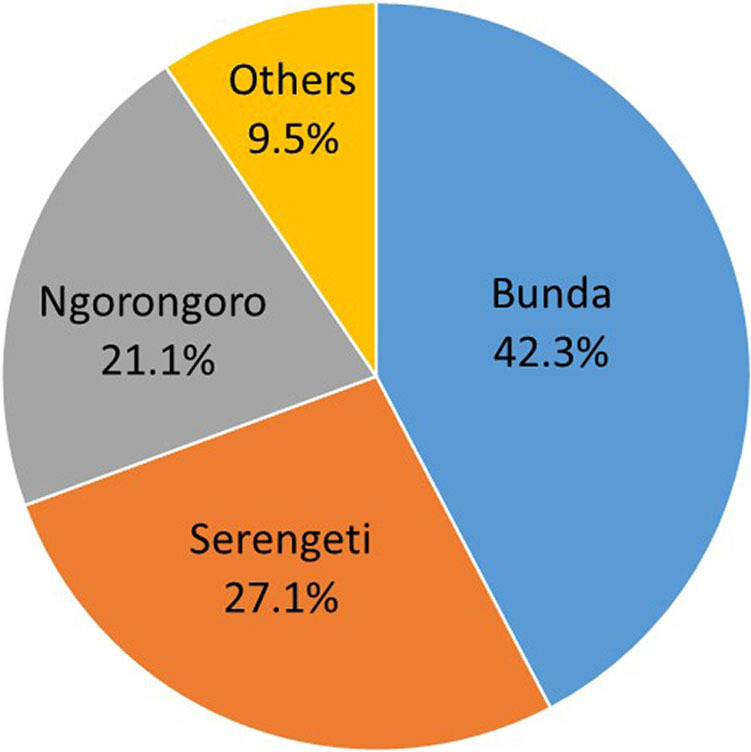
Distribution of Presumptive TB Patients Participating in the Study by District (N=421)

The communities living in the Serengeti ecosystem consist of a heterogeneous mixture of ethnic groups who have migrated from various regions within Tanzania and who pursue a variety of economic activities with a common language, Swahili. A large proportion of presumptive TB patients were found in Bunda (42.3%), compared with Ngorongoro (21.1%), Serengeti (27.1%), and other districts (9.5%) (Figure 3). In the Serengeti and Ngorongoro districts, most presumptive TB patients came from communities involved largely with farming (ie, growing crops and keeping livestock), whereas Bunda consisted of a more heterogeneous population including farmers as well as traders, tailors, and fishermen. Some patients who visited the district-designated hospital in Bunda, however, came from villages outside of Bunda district, such as from Lamadi in the Magu district of Mwanza alongside the Serengeti National Park, and also from Musoma and other nearby districts of Mara. Therefore, data from the Bunda district-designated also represents residents from other nearby districts within Mara and just outside the region. The location of the Bunda district-designated hospital makes it accessible from every corner of the ecosystem.

### Data Analysis

**FIGURE 4.**
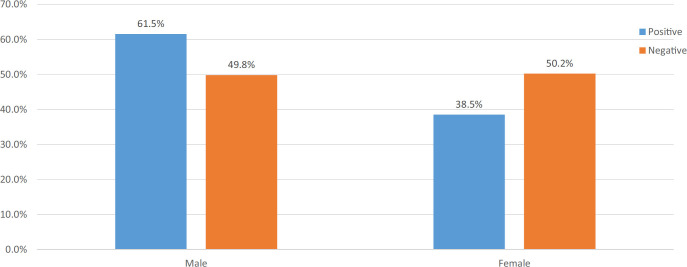
Distribution of Presumptive Tuberculosis Patients Participating in the Study by Gender (N=421)

Sputum samples were obtained from 421 presumptive TB individuals collected over a 1-year period. Gender representation was almost equal. These samples were subjected to mycobacterial culture, of which 187 tested positive for *M tuberculosis*, or 44% (95% CI, 0.40–0.49) of all collected samples. None of the sputum culture growths consisted of *M bovis* or non-tuberculous mycobacteria. Comparison between gender and TB status indicated a significant difference, with males being more frequently culture positive for the disease compared with females (*χ*^2^=5.7, df=1, *P*=.017) (Figure 4). Antibiotic sensitivity tests showed no phenotypic resistance with a standard minimal inhibitory concentration in Löwenstein-Jensen medium to isoniazid, rifampicin, ethambutol, or streptomycin.

The culture results of TB patients by age are shown in Table 1. Most TB-positive patients were between 20 and 39 years old. The frequency of distribution was skewed toward older ages within this age group, with a statistically significant association between TB culture positivity (TB status) and age (*χ*^2^=14.1, df=4, *P*=.007). The proportion of TB-positive (45%) and TB-negative (55%) patients did not reflect much difference.

**TABLE 1.** Tuberculosis Status of Culture-Tested Participants by Age (N=416)

	Tuberculosis Culture		
Age	Positive (%)	Negative (%)	Total (%)
No category	2 (1.1)	1 (0.4)	3 (0.7)
<20 yrs	10 (5.3)	29 (12.7)	39 (9.4)
20–39 yrs	104 (55.6)	91 (39.7)	195 (46.9)
40–59 yrs	51 (27.3)	75 (32.8)	126 (30.3)
60 yrs and above	20 (10.7)	33 (14.4)	53 (12.7)
*Total*	*187 (45.0)*	*229 (55.0)*	*416 (100.0)*

Of all participants analysed for culture results, 291 (71.9%) were married, 89 (22%) were unmarried, 20 (4.9%) were widowed, and 5 (1.2%) were divorced. Of the culture-positive TB patients, married participants accounted for 69.3% of the group followed by unmarried participants (22.9%). Smear-positive and culture-positive participants who had a previous history of BCG vaccination (65, 34.8%) compared with those who had no previous history (114, 61.5%) shows clear evidence that BCG vaccination protected individuals against TB by nearly twofold, indicating that the 2 groups differed significantly in potential susceptibility to TB (*χ*^2^=14.9, df=2, *P*=.001). Of the participants who tested positive for TB based on smear and culture samples, 61.5% had no BCG vaccination history, 34.8% had a history of vaccination against TB, and 3.7% did not know their childhood BCG vaccination status. Analysis of other infections associated with TB among participants revealed that 21 (49%) had known cases of HIV infection and 22 (51%) had other associated infections or conditions including wounds and skin rashes.

### TB Test Results and Occupation

The Serengeti ecosystem is predominantly inhabited by pastoralists, although many community members engage in other livelihood activities. Pastoralists keep animals and move around the ecosystem in search of better pastures when grazing becomes limited, particularly during the dry season. Our results (Table 2) showed that the majority of TB culture-positive participants (61.5%) were farmers who raised livestock and crops. The proportion of those farmers who kept livestock only (15%) was the second largest group of TB culture-positive participants, followed by the group designated “others” that included street vendors, tailors, plumbers, masonry workers, welders, and bar maids (12.3%). The percentage of the latter group was greater than the percentage of culture-positive students and traders combined (11.2%). Pearson Chi-square group comparisons showed an association between the proportion of TB-positive patients and their occupation (*χ*^2^=10.3, df=4, *P*=.04), reflecting the impact occupation may have on infection status.

**TABLE 2.** Tuberculosis (TB) Status of Culture-Tested Participants by Occupation, Previous Contact With TB Patients, and Window Size (N=416)

		TB Status		
Variable	Variable Category	Positive (%)	Negative (%)	Total (%)
Occupation	Farmers (livestock and crops)	115 (65.1)	111 (85.5)	226 (54.3)
	Farmers (livestock only)	28 (15)	55 (24)	83 (20)
	Students	10 (5.3)	23 (10)	33 (7.9)
	Traders	11 (5.9)	13 (5.7)	24 (5.8)
	Others^a^	23 (12.3)	27 (11.8)	50 (10)
Previous contact with TB patients^b^	No	112 (59.9)	119 (52)	231 (55.5)
	Yes	38 (20.3)	58 (25.3)	96 (23.1)
	Not known	37 (19.8)	52 (22.7)	89 (21.4)
Window size	Small	101 (54.0)	105 (47.1)	206 (50.2)
	Medium	68 (36.4)	106 (47.5)	174 (42.4)
	Large	18 (9.6)	12 (5.4)	30 (7.3)

a Includes fishermen, tailors, plumbers, carpenters, and street vendors who were found in small proportions.

b Refers to contact with TB patients in the previous 12 months.

### Knowledge About TB and TB Status

Nearly all (92%) of the participants in this study had prior knowledge about how TB infection can be acquired and transmitted from an infection source. As reflected in Table 2, previous contact with a TB patient was not significantly associated with TB infection, as only 38 (20.3%) of TB culture-positive participants had known previous contact with TB patients in the last 12 months before testing themselves. In addition, among those participants who had previous contact with TB patients, 16 (42.1%) had contact with patients aged 40 to 59 years.

### Culture Results and Housing

Most TB culture-positive participants lived in houses constructed with iron sheets (63.1%), mud walls (62.4%), 3 to 5 rooms (59.1%), and 3 to 5 windows (57.8%). Most participants resided in houses with small windows (54%), and the remainder (36.4% and 9.6%) had medium and large windows, respectively (Table 2). In contrast, most (53%) TB culture-negative participants lived in dwellings with medium to large windows. Thus, window size seems to be associated with TB risk (*χ*^2^=6.5, df=2, *P*=.04), particularly small windows. The latter group of culture-positive patients who lived in houses with small windows also lived with 2 to 5 other people (163/187, 87.2%). Very few patients who tested positive for TB came from single-person households (3.2%) or more than 6 members per household (9.6%). There was no evidence that sleeping in a household with livestock was associated with testing positive for TB (*χ*^2^=9.8, df=1, *P*=.002). This is indicated by the results that a higher proportion (88.2%) of TB-positive patients had never slept with livestock in the same household, and only 11.8% habitually slept with livestock in the same household.

### Cigarette Smoking, Alcohol Use, and Social Networking

The results also revealed that 85% of the participants who tested positive for TB were non-smokers, providing evidence that cigarette smoking alone might not be a major risk factor for developing disease in this population (*χ*^2^=10, df=1, *P*=.002), and 85% also did not drink alcohol. In other words, non-smokers were more likely to have TB compared with smokers. Among smokers, however, the majority were TB positive; of all smokers (n=41), 28 (68.3%) had positive TB culture results compared with 13 (31.7%) who were culture negative. This means that if we had tested for TB in smokers only, more smokers could test positive than negative. Similarly, the effect of drinking alcohol was such that not drinking alcohol was associated with being TB negative, whereas drinking alcohol was associated with being TB positive (*χ*^2^=6.9, df=1, *P*=.01).

### Quantification of Risk Factors for TB Occurrence

The data were subjected to multivariable logistic regression to quantify the contribution of different factors to TB occurrence. The final logistic regression model had 5 explanatory variables as shown in Table 3. A total of 74 observations were excluded due to missing values. The Hosmer and Lemeshow goodness-of-fit test yielded a *P* value of .72. Contact with livestock products, infrequency of milk consumption, cigarette smoking, and alcohol consumption were the main risk factors for occurrence of TB, whereas recent contact with livestock (within the past 12 months) was found to be protective (Table 3). People in contact with livestock products were 6 times more likely to have TB infection compared with those not in contact. On the other hand, the risk of becoming infected with TB was 2.9 (95% CI, 1.19–7.12) times higher for cigarette smokers and 2.3 (95% CI, 1.22–4.23) times higher for consumers of alcohol compared with those who did not smoke or drink. Habitual milk drinking in one way or the other affected occurrence of TB in the community. Frequency of milk consumption, for example, increased risk of *M tuberculosis* infection by 2.5 times for people who did not consume milk on a daily basis compared with people who drank milk at least once a day (Table 3). There were neither confounders nor significant interactions between explanatory variables in the final model.

**TABLE 3.** Explanatory Variables as Potential TB Risk Factors

Variable	Response	Frequency (*n*=347)	% TB Case (*n*=148)	OR	95% CI
Recent contact with livestock (within the past 12 months)	No (0)	160	61.6	1.0	0.25–0.75
	Yes (1)	185	38.4	0.43		
Contact with livestock products	No (0)	20	2.7	1.0	1.81–19.9
	Yes (1)	327	97.3	6.0	
Milk consumption	At least daily (0)	171	33.0	1.0	1.42–4.23
	Not daily (1)	176	67.0	2.5	
Smoke cigarettes	No (0)	315	84.0	1.0	1.19–7.12
	Yes (1)	32	16.0	2.9	
Drink alcohol	No (0)	278	73.0	1.0	1.22–4.23
	Yes (1)	69	27.0	2.3	

Abbreviations: CI, confidence interval; OR, odds ratio; TB, tuberculosis.

## DISCUSSION

The participants in this study represent a heterogeneous community of farmers (growing crops and keeping livestock), livestock keepers (who keep only livestock), traders, and other people who engage in formal and non-formal livelihood activities around the Serengeti ecosystem. The prevalence of TB in this area in humans and cattle is 0.2%^1^ and 2.4%, respectively.^11^ It is highly likely that these groups of people have different levels of exposure to the disease and therefore variability in incidence rates.

Previous studies have indicated the association between age and TB infection with a change in prevalence by age^18–21^ because the disease is chronic in nature.^1^ This study found more TB culture-positive patients between 20 and 39 years old, potentially the most economically active and productive age group. TB affects mostly young adults and is among the top 3 causes of death for women ages 15 to 44 years, although all age groups are at risk.^1,22^ Being in this age range could also be a major risk factor for other infections, particularly in Bunda where contact and interaction between people is very high due to location and access to road infrastructure and connections to other places within the region. Married participants accounted for 69.3% of TB-positive cases, signifying high transmission of TB within this group.

The relationship between HIV and TB in this study can be found in a separate report by Mbugi and colleagues.^23^ Interestingly, and probably not surprising, is that HIV accounted for 21 (49%) of other infections active in TB-positive individuals. Previous studies reported more than 30% TB-HIV coinfection,^24^ and up to almost 90% TB-HIV coinfection^25^ in some parts of Africa. The updated World Health Organization (WHO) report indicates 13% TB-HIV coinfections.^1^ More reports address concerns on TB-HIV coin-fection,^26–29^ despite one study reporting a lack of concrete TB-HIV coinfection in children.^30^ In regions where TB and HIV/AIDS are endemic, particularly sub-Saharan Africa, people living with HIV are nearly 20 times more likely to develop TB compared with those who are HIV negative.^24^ This is particularly important as HIV plays a role in compromising the immune response to TB,^28^ although the mechanisms by which HIV disrupts TB immune pathology are unclear.^31^ Our findings are very similar to findings from Piggott and Karakousis, which reported more than 50% active TB-HIV coinfection.^32^ Infection with HIV is known to predispose the host to *M tuberculosis* latent infection and progression to active disease, which increases the risk of latent TB reactivation by 20-fold.^27^ The role HIV plays in increasing susceptibility to TB is clear, particularly through reduction in CD4+ T cells count and function.

### TB Test Results and Occupation

Study results showed that TB infection was much more prevalent in farmers (Table 2), but this should not be misinterpreted to mean that other groups (students, traders, and others—street vendors, tailors, plumbers, masonry workers, welders, and bar maids) are not at risk. The data suggest that the disease can affect all groups regardless of occupation and age. The study showed that 5.3% of TB-positive cases were students at different stages of school life. This is critical because one would expect the younger generation at school to be healthy. The results of this study imply that special attention should be paid to students to protect them from chronic infections that could jeopardise their school performance and future development. The results of this study do not explain why TB infection was more prevalent among farmers or the association between TB infection and occupation in general. The variability in infection rates by occupation could be attributed to lifestyle and other factors not associated with human-animal interface.

### TB Test Results and Keeping Animals

The study findings reflected that TB infection is determined by a combination of factors rather than a single factor. Keeping cattle plays a key role in becoming infected with bovine TB (bTB), especially the type and frequency of contact with infected livestock and their products. Other studies also show evidence of transmission of TB from humans to animals and vice versa, particularly in elephant farms.^33,34^ Recent conversations with a Keystone delegate confirm that humans in the United States and Canada have transmitted TB to cattle (unattributed). One report, however, suggests that transmission of TB from cattle to humans is still questionable, as the contribution of *M bovis* to human TB is considered minor.^6^ Our study failed to establish the causal relationship because of non-detection of *M tuberculosis* in animals.^12,17^ Transmission of bTB to humans is thought to occur through consumption of livestock products, predominantly milk but also meat.^35^ Whether the transmission of bTB to humans constitutes a public health concern remains a question. In this study, descriptive analyses indicated no evidence that drinking milk, boiling milk before drinking, or drinking either sour or fresh milk were major risk factors associated with TB infection (an association would have been more likely if patients were infected with *M bovis*). However, results from the logistic regression analysis suggested that a combination of multiple factors may play a role in increasing risk for TB infection (Table 3). For example, frequency of milk consumption revealed a 3.7 times higher likelihood of TB infection for people who did not consume milk on a daily basis compared with people who consumed milk at least once a day. The higher risk in this group might be caused by a confounding factor, for example, people who consume milk less frequently could have a different lifestyle that increases their chances of becoming infected with TB.

### Knowledge About TB and TB Status

The majority of patients tested in this study (91.8%) had knowledge about TB infection and factors associated with a positive sputum smear test; 95.7% who tested positive for TB had at least some idea of how TB can be contracted and transmitted. This high level of knowledge implies that other unknown factors are likely playing a role in transmission, for example, environmental contamination through overcrowding in transport vessels. Level of education and frequency of awareness campaigns by credible sources might play a crucial role in reducing TB incidence.

TB is a chronic infection that can be latent over a long period of time, and therefore asymptomatic TB carriers can unknowingly pass it on to uninfected individuals. This is especially threatening if the carrier possesses drug-resistant TB, in particular multidrug-resistant TB or extensively drug-resistant TB. It has been suggested^35^ that despite the potentially minor contribution of bTB in human TB, there is a critical need for interventions to control the disease and prevent zoonotic transmission of *M bovis* to human populations consuming dairy products. As reflected in this study, people in contact with livestock products were 5.4 times more likely to become infected with TB compared with those not in contact with livestock (Table 3). The risk of TB transmission is bidirectional (human to animal and animal to human), as indicated by reports on transmission from humans to cattle^36,37^ and dogs,^38^ which reflect the importance of zoonotic transmission of TB in the Serengeti ecosystem communities.

Education, socioeconomic factors, and living standards may play important roles in reducing transmission among pastoralists. Analysing such factors to determine ways to reduce transmission will be particularly critical to prevent the spread of multidrug-resistant TB in the region.

### Culture Test Results and Housing

This study evaluated the construction material of housing and living standards and found that TB culture-positive participants came from houses thatched with iron sheets (63.1%), constructed with mud-made walls (62.4%), and having 3 to 5 rooms (59.1%) and 3 to 5 windows (57.8%) per house. These findings are contrary to previous findings that support the well-known relationship between poverty, disease, poor indoor ventilation, and transmission of disease. The houses of participants in this study seemed to have space; however, the number of rooms may have led to many people living in one household, thus predisposing them to overcrowding—a risk for disease transmission from infected to healthy individuals. Potential overcrowding would be exacerbated by the fact that a large proportion of TB culture-positive participants lived in houses with small windows (Table 2). The combination of small windows and overcrowding could predispose patients to infection due to the aerosol nature of transmission of the disease. A recent report^39^ indicates a higher risk and predisposition to TB transmission in an environment with limited air flow and low rates of ventilation in households. Multiple factors, however, contribute to airborne pathogen transmission from infectious carriers, including cough frequency, respiratory rate, and duration of exposure between source and contact.^40^

The houses lived in by study participants also had at least 2 to 5 members (87.2%) per household, further signalling the potential impact that overcrowding may have on disease transmission. Environment is an important factor in TB transmission^41^ and should not be overlooked. Our study also showed that very few TB culture-positive patients were from single-occupancy households (3.2%); this finding aligns with the principles of airborne disease transmission. The risk of contracting a disease increases as the number of occupants per unit room increases. Thus, the increased risk among 163 (87.2%) patients who slept in households that were occupied by 2 to 5 other members is justified.

The evidence for transmission of TB infection from humans to animals is available^42,43^ but rarely from animals to humans. We also did not find any association between TB incidence and sleeping in a household with livestock. Most TB-positive participants (88.2%) never slept in a household with animals and only 11.8% originated from a household with animals. The results are predictable, as there were no positive results of *M bovis* in TB-positive participants.

### Cigarette Smoking, Alcohol Use, and Social Networking

Our study did not show any association between cigarette smoking or alcohol use and smear-positive TB, indicating that cigarette or alcohol use alone might not be major factors for developing the disease, but a combination of factors might contribute. Visits to gatherings (ie, social networking) had no direct association with culture-positive TB. However, logistic regression analysis revealed that cigarette smokers and consumers of alcohol are at risk of becoming infected with TB (3 and 2 times more likely) compared with non-smokers and non-drinkers alone. The association between TB and smoking has been previously reported as having a strong dose-response relationship, both in terms of quantity and duration of smoking.^44–46^ Smoking increases workload to the lungs and is said to potentially decrease the immune response or damage the function of cilia in the airways.^47^ It is also reported that cigarettes contain nicotine, which exerts immunosuppressive effects on immune surveillance through functional impairment of the dendritic cell system.^48^ By doing so, the ability of immune cells to induce differentiation and expansion of type 1 T immune cells is reduced, thus decreasing the frequency of interferon-*γ*-producing effector cells for body defence. In such situations, the risk for TB increases due to diminished protection.

With patients in this study coming from families of different economic status, our objective should focus on alleviating these communities from poverty to improve their economic status, which is the key toward solving health problems related to infectious diseases. Tuberculosis is a disease of poverty, and therefore a solution to poverty could help reduce the impact of the disease. Only by successfully containing the disease can we realise the epidemiological and economic projections of averted mortality and economic benefits in sub-Saharan Africa and other high-burden, TB-endemic countries.^49^ Education to these communities on the One Health approach in tackling zoonotic infections like TB, combined with a participatory approach from professional and non-professional stakeholders, could play a key role in this context. Joint efforts could have a big impact in finding solutions for cross-cutting infectious diseases in resource-constrained countries, most of them found in Africa. For example, some studies in Africa have called for government and policy makers to work together with other stakeholders to design methods that could control bTB in intensive farming communities.^35^ In so doing, any transmission of bTB to humans will be prevented, particularly if contact investigation for early case detection is combined with treating latent TB infection.^50^ This study focused on analysing some of the risk factors (eg, milk consumption and occupations involving livestock) to identify potential associations with zoonotic transmission of TB. However, the culture results showed no evidence of zoonotic transmission (*M bovis* growth), disproving the idea that zoonotic transmission was a major risk factor in this population.

## CONCLUSION AND RECOMMENDATIONS

There was no evidence of direct cross-species transmission of either *M tuberculosis* or *M bovis* between humans and animals using the study methods. A parallel study that tested wildlife and livestock tissues^12^ also showed no evidence of *M tuberculosis* in cattle and wildlife, further limiting the possibility of TB cross-transmission between these species.

The absence of *M bovis* in humans and *M tuberculosis* in animals does not, however, rule out potential cross-species transmission if the infection happens to be in either of the hosts in these endemic areas. The absence of evidence of cross-transmission could be due to limited chances of contact rather than absence of transmission between these species. Our future plans include tracing households with TB-positive patients and concomitant infection in animals to enable identifying the virulence gene in both *M tuberculosis* and *M bovis* and their probable differences. Precautionary measures are therefore still required in farming communities where animal-human interaction is intense.

Our statistical analysis revealed that recent contact with livestock is a potentially protective factor, similar to frequent milk consumption. We interpret this finding with caution, however, as contact with livestock products such as milk and meat might also be a risk in case of bovine TB transmission in the ecosystem. We also found an association between infrequent milk consumption and TB infection. Although this finding seems counterintuitive and may be attributed to chance, it is important to acknowledge its potential contribution.

This study found a higher rate of TB infection among participants between 20 and 39 years old, potentially the most economically active and productive age group, and also prone to HIV/AIDS. HIV impairs the immune system and can increase the risk of active TB, further contributing to the spread of TB. WHO^51^ reports that people infected with HIV are 21 to 34 times more likely to become infected with TB.

This study found that a combination of factors is associated with TB infection—contact with livestock products, infrequent milk consumption, cigarette smoking, and alcohol consumption. These factors may influence the dynamics and impact of the disease in the Serengeti ecosystem.

From this study we can also learn that not all people with presumptive TB are infected with TB, and therefore control strategies should emphasise confirming TB status before administering anti-TB drugs.
